# Enhancing Interfacial and Mechanical Properties of Carbon Fiber/Poly (Ether Ether Ketone) Composites via Bisphenol-Based Polyimide Modification

**DOI:** 10.3390/polym17243258

**Published:** 2025-12-07

**Authors:** Aylin Albayrak, Mustafa Dogu, Mustafa Cakir, Kadir Turhan

**Affiliations:** 1 Department of Chemistry, Faculty of Arts and Sciences, Yildiz Technical University, Davutpasa Campus, Esenler, 34220 Istanbul, Türkiye; kturhan@yildiz.edu.tr; 2 Research Department, Mir Arastirma ve Gelistirme Inc., Esenyurt, 34220 Istanbul, Türkiye; mus.dogu@gmail.com; 3 Department of Materials and Metallurgical Engineering, Marmara University, Maltepe, 34854 Istanbul, Türkiye; mcakir@marmara.edu.tr

**Keywords:** carbon fiber composites, bisphenol-based monomers, polyimide sizing, poly (ether ether ketone), interfacial adhesion

## Abstract

This study focuses on the synthesis of two new bisphenol-based polyimide (PI) sizing agents to improve the fiber–matrix interface of carbon fiber-reinforced poly (ether ether ketone) (CF/PEEK) composites. One of the synthesized polyimides contains bisphenol A (BPA) monomer, while the other has bisphenol S (BPS) monomer. The produced polyimide precursor resins were coated with carbon fibers by thermal imidization. The thermal, thermomechanical, and mechanical properties of the CF/PEEK composites produced by the autoclave process were investigated. According to the mechanical test results, there was a balanced performance between the BPS-containing polyimide-coated composites (CF-PEEK-PI-S) and the BPA-containing polyimide-coated composites (CF-PEEK-PI-A). While tensile strength is 291 MPa and interlaminar shear (ILSS) strength is 119 MPa, the CF-PEEK-PI-A sample showed superior mechanical properties in flexural (92.1 MPa) and compressive strength (54.9 MPa). As a result, it was concluded that bisphenol-based polyimide coatings significantly improve the interfacial interactions in CF/PEEK composites, which have great potential in aerospace, automotive and advanced engineering applications.

## 1. Introduction

Thermoplastic composites have gained increasing attention in aerospace, automotive, and fiber-reinforced materials, and have become preferred over thermoset composites due to their excellent material strength and recyclability [1,2,3,4,5]. In particular, carbon fiber (CF), a widely preferred material in high-performance composites, is a sought-after reinforcement element due to its properties, such as high corrosion resistance and excellent strength-to-weight ratio [6].

Poly (ether ether ketone) (PEEK) is a high-performance thermoplastic widely used as a matrix element in composite materials [7,8]. PEEK offers outstanding mechanical properties, a high melting point of approximately 340 °C, and excellent flame retardancy due to its aromatic structure [9,10,11,12,13]. Despite its advantageous properties, its very high melting point presents technical challenges during fiber processing [14,15,16,17]. Furthermore, the nonpolar structure of CF shows low wettability and weak interfacial bonding when combined with PEEK [18,19,20,21].

Various surface functionalization methods are applied to the carbon fiber surface to strengthen the fiber–matrix interface and enhance performance [22] such as ultrasonic treatment [23], plasma activation, oxidative surface treatments [24] and sizing [24,25,26,27]. The sizing method is particularly prominent due to its high efficiency and practicality [28].

Polyimides (PIs) are widely used among the sizing agents because of their superior properties. Their high aromatic group content gives them excellent thermal stability and mechanical strength in fiber-reinforced composites for high-performance applications such as the aerospace and automotive industries [2,29]. PIs are synthesized in a two-step procedure by reacting a diamine with a 1:1 ratio of dianhydride [30]. The first step involves dissolving the polyimide precursor in polar aprotic solvents at room temperature. The viscous product obtained at this stage is called a polyamic acid (PAA). The second step involves cyclization of the amic acid groups to form the imide ring and removal of water to form the polyimide [31].

There are many studies on polyimide to improve the bond between CF and PEEK composite materials. Ref. [32] reported the use of a PAA sizing agent, free of organic solvents, to improve the chemical interaction between polyether sulfone (PES) composites and CFs. The results were quite satisfactory. Compared with commercially available CF, the interfacial shear strength (IFSS) of CF/PES composites increased by 47.9%, from 33.6 to 49.7 MPa. They reported that the synthesized new sizing agent significantly improved the surface energy of the fibers, although it did not significantly alter their surface morphology. Ref. [33] synthesized PAA to improve the interfacial performance of carbon fibers and matrices. They used aromatic bisphenol A dianhydride (BPADA) and 4,4-oxydianiline (ODA) as monomers. They also produced a composite material composed of carbon nanotubes (CNTs) for comparison. They reported that the bare composite (CF/PI) achieved a flexural strength of 420 MPa, while the CF/PI composite treated with CNTs achieved a flexural strength of 560 MPa. In one of the other studies, refs. [34,35] prepared a polyimide sizing agent reinforced with carbon nanotubes. They modified this sizing material to fit CF. As a result of this modification, the flexural and interlaminar shear strengths of CF/PEEK composites increased by 63.2% and 70.5%, respectively. Thus, they reported a sizing agent that improved the fiber surface properties such as wettability and polarity.

The current work focuses on the synthesis of two different bisphenol-based PI sizing agents to improve between CF and PEEK. One of the PIs contains bisphenol-A (BPA) diamine, and the other has bisphenol-S (BPS) diamine. The novelty of this work is the development of new PI sizing materials based on bisphenol chemistry, which has not been widely explored at CF/PEEK interfaces. The aim of present study is to compare the thermal, mechanical, and thermomechanical performance of PIs synthesized from two new bisphenol-based diamines, instead of the diamines commonly used in commercial PI synthesis, at the CF/PEEK interface.

## 2. Materials and Methods

### 2.1. Materials

For all experimental investigations, Hexcel Corporation’s Hex Force AGP280-5 H 5HS (Stamford, USA) woven carbon fabric was selected as the reinforcing textile in the preparation of polyimide-based systems. The monomers were obtained in high purity: bisphenol A (99%), bisphenol S (99%), pyromellitic dianhydride (97%), and 1-chloro-4-nitrobenzene (98%) were supplied by Thermo Fisher Scientific (Dreieich, Germany). Other chemicals, namely potassium carbonate (99%) and 10% palladium on carbon, were provided by Sigma-Aldrich (Schnelldorf, Germany). Hydrazine monohydrate (65%) (Sigma-Aldrich, Schnelldorf, Germany) was employed as a reducing agent. Dimethylformamide (99%) and N-methyl-2-pyrrolidone (99%), (Sigma-Aldrich, Schnelldorf, Germany)functioned as solvents during the synthesis. Ethanol (99%) (Sigma-Aldrich, Schnelldorf, Germany) was further used in recrystallization. The PEEK polymer powder served as the matrix was bought from Evonik Industries, Dusseldorf, Germany

### 2.2. Monomer Synthesis

#### 2.2.1. 4,4′-(Sulfonylbis (4-nitrophenoxy) benzene) (DNPSB)

**Figure 1:**
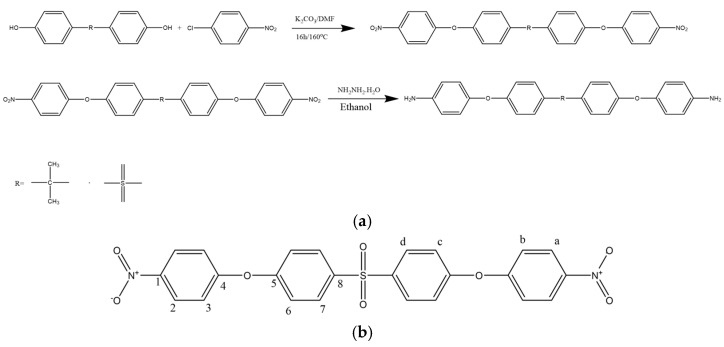
Reaction Scheme for Synthesis of Diamine (**a**) and ^1^H NMR and ^13^C NMR of DNPSB (**b**).

All the reactants (0.075 mol of bisphenol S, 0.16 mol of 1-chloro-4-nitrobenzene, and 300 mL of DMF) were placed in a three-necked balloon. After it was dissolved, 0.16 mol of potassium carbonate was added to the mixture. The mixture was refluxed at 160 °C under a nitrogen atmosphere for approximately 16 h. After 16 h, the mixture was poured into ice water. The resulting precipitate was collected by filtration. The crude dinitro compound was dried under vacuum and then recrystallized from ethanol to obtain a yellow solid in 60% yield. The diamine monomer was synthesized in a two-step reaction as shown in Figure 1a,b. mp: 186–188 °C.

#### 2.2.2. 4,4′-((Sulfonylbis (4,1-phenylene)) bis(oxy)) dianiline (BAPS)

**Figure 2:**
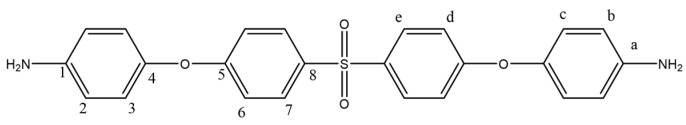
^1^H NMR and ^13^C NMR of BAPS.

To obtain the diamine product in the second step of the reaction, 25 mL of hydrazine (65%) was added dropwise to a mixture containing 0.05 g of 10% Pd/C and 0.02 mol of the dinitrogen compound in the presence of ethanol. The mixture was stirred in a reflux condenser under nitrogen gas for approximately 12 h. After completion, following filtration, the resulting precipitate was recrystallized in ethanol and dried in a vacuum oven at 40 °C. A yellow diamine compound was obtained in 65% yield (Figure 2). The synthesis route of the diamine monomer was adapted from a literature procedure [36]. mp: 184–185 °C.

#### 2.2.3. 4,4′-((Propane-2,2-diyl) bis(4-nitrophenoxy) benzene) (BPA-DN)

**Figure 3:**
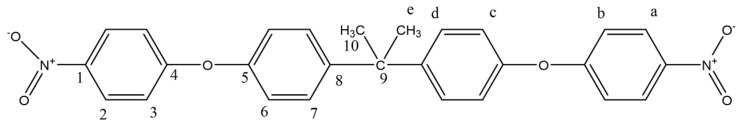
^1^H NMR and ^13^C NMR of BPA-DN.

The reaction was the same as that of the BPS-based compound. Briefly, 0.075 mol BPA, 0.16 mol 1-chloro-4-nitrobenzene, 0.16 mol potassium carbonate, and 300 mL DMF were placed in a 3-neck flask with a nitrogen inlet and a reflux condenser. It was refluxed at 160 °C under a N_2_ atmosphere for 16 h. After the reaction was completed, the mixture was dropped into an ice–water mixture. The precipitate, collected by filtration, was dried in a vacuum oven and then crystallized in ethanol. The yellow dinitro compound was obtained in 60% yield (Figure 3). mp: 123–124 °C.

#### 2.2.4. 4,4′-((Propane-2,2-diylbis(4,1-phenylene)) bis(oxy)) dianiline (BAPP)

**Figure 4:**
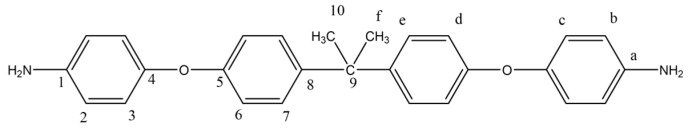
^1^H NMR and ^13^C NMR of BAPP.

In the second step of the reaction, 0.05 mol of the dinitrogen compound was reacted at 85 °C in the presence of ethanol under reflux with the addition of 0.1 g of 10% Pd/C. 55 mL of hydrazine (65%) was added dropwise. Approximately 12 h later, the precipitate obtained by filtration process was recrystallized in ethanol. The yellow diamine compound was obtained with 70% yield by drying in a vacuum oven at 40 °C (Figure 4). The synthesis route of the diamine monomer was adapted from a literature procedure [37]. mp: 132–134 °C.

#### 2.2.5. Synthesis of PAA Solution and Coating of Carbon Fibers

Different chemical structures of PAAs were formed using commercially purchased pyromellitic dianhydride (PMDA) and two different synthesized diamine monomers. For this purpose, the diamine and diamine monomers reacted at 1:1 weight ratio in a three-necked flask under reflux in the presence of NMP solvent. The reaction mixture (approximately 40 mL) was stirred at room temperature under a N_2_ atmosphere, and after 16 h, a viscous PAA solution was obtained. The solution was then homogenized with a suitable solvent to form a 15% ratio of each resin.

The obtained PAA resins and PIs are shown in Table 1.

The purchased CF was cut to 35 × 35 cm. Commercially available CFs are coated with an epoxy-based sizing agent [38,39]. First, the epoxy sizing was removed using an infrared lamp (IR) for a short period to coat the fiber with newly synthesized sizing agents. The removal process was monitored visually to ensure that the residual commercial sizing agent had been removed, with the fiber surface losing its glossy appearance. The bare fibers, which lacked sizing agents, were then dipped into homogeneous resins and coated. They were then incubated in a 250 °C oven for 3 h. The amic acid rings, subjected to thermal imidization, transform into a cyclic structure at high temperatures, water is removed, and the polyimide is coated onto the carbon fibers.

#### 2.2.6. Coating of CFs with PEEK and Fabrication of Composite Materials

Fibers coated with a sizing agent were then subjected to a powder polymer dispersion unit and an IR source with medium-wavelength lamps to melt the polymer into the fiber. This allowed the PEEK polymers to be impregnated into the fibers, creating prepregs. Eight layers of prepregs were stacked and subjected to an autoclave process using vacuum bag support.

#### 2.2.7. Characterization

##### Chemical Characterization

FT-IR and Fourier Transform Infrared Spectroscopy (using a Thermo Scientific Nicolet iS10 (Thermo Fischer Scientific, Waltham, MA, USA)) were used to confirm the presence of –NO_2_ and –NH functional groups in the synthesized diamine monomers. The purity control and chemical structure verification were performed using a Bruker Avance NEO 500 MHz Nuclear Magnetic Resonance (NMR) (Bruker Corporation, Fällanden, Switzerland) instrument.

##### Thermal Analysis

To determine the melting points of monomers, resins, and composite materials were used a Perkin Elmer DSC 4000 Differential Scanning Calorimetry (DSC) (PerkinElmer Inc., Waltham, MA, USA) instrument. DSC measurements were performed under nitrogen atmosphere at a heating rate of 20 °C/min in the range of 30–375 °C. Samples were cooled to 30 °C at 10 °C/min and reheated at 20 °C/min for the second heating cycle. Thermal analyses of resins and produced materials were performed using a TA Instruments Q50 Thermogravimetric Analysis (TGA) (TA Instruments, New Castle, DE, USA) instrument. Analysis was performed from room temperature to 800 °C at a heating rate of 10 °C per minute under a nitrogen atmosphere. Dynamic Mechanical Analysis (DMA) of composite materials was determined a TA Instruments DMA Q800 model (TA Instruments, New Castle, DE, USA) machine. Sample dimensions were 10 mm and 35 mm in width and length, and the tensile frequency was set at 1 Hz. Tests were conducted at temperatures between 35 °C and 250 °C with a heating rate of 5 °C per minute.

##### Surface Morphology

The surface morphologies of the composites were examined with two Scanning Electron Microscope (SEM) devices. SEM analysis of bare CF composite (was performed on a Zeiss EVO LS 10 SEM (Carl Zeiss Microscopy GmbH, Oberkochen, Germany)device at Istanbul Yıldız Technical University. Other SEM analysis was performed using an Oxford Instruments S3 microscope (Oxford Instruments, Oxford, United Kingdom) at the University of Warwick.

##### Mechanical Tests

The Zwick Z250 Universal Testing Machine was used for the mechanical strength tests of the composites. Tensile tests were carried out according to the ISO 527-4 standard [40], with length-to-width ratio set at 250 mm and 25 mm, respectively, at a speed of 2 mm/min. Flexural tests were conducted according to EN 2562 [41]. The materials had a length-to-width ratio of 60 mm and 10 mm, and the tests were conducted at a speed of 5 mm/min. Compressive tests were conducted according to ASTM D3410 [42], with lengths and widths of 145 mm and 25 mm, respectively, and the tests were conducted at a speed of 1.5 mm/min. Five parallel specimens were tested for each mechanical experiment, and the results are presented.

##### Interlaminar Shear Strength (ILSS)

The interlaminar shear strength (ILSS) test was determined according to EN 2563 [43]. The materials had a length-to-width ratio of 20 mm and 10 mm, and the test speed was set at 1 mm/min. Six parallel specimens were tested for ILSS test, and the results are presented.

## 3. Results

### 3.1. Monomer Synthesis

In this study, nucleophilic aromatic substitution reactions were conducted with p-chloronitrobenzene using both bisphenol-S and bisphenol-A compounds. -NO_2_, a strong electron-withdrawing group in the reaction, increased the reaction efficiency by facilitating the removal of chlorine. In the second step of the reaction, a Pd/C catalyst and hydrazine hydrate were used to reduce the dinitro compounds. During this reduction, the -NO_2_ groups on the aromatic ring were converted to -NH_2_ groups. This made the reaction occur with high yield and selectivity. FTIR, DSC, ^1^H NMR and ^13^C NMR spectra confirm the purity and accuracy of the compounds that have characteristic peaks/bands (Supporting Information).

#### 3.1.1. Structural Characterization of Monomers

##### 4,4′-(Sulfonylbis (4-nitrophenoxy) benzene) (DNPSB)

IR (KBr, cm^−1^): 1340, 1514 (-NO_2_ stretching), 1239 (C-O-C stretching). ^1^H NMR (500 MHz, DMSO-d_6_): 8.27 (4H, H_a_), 7.21 (4H, H_b_), 7.27 (4H, H_c_), 8.15 (4H, H_d_). ^13^C NMR (500 MHz, DMSO-d_6_): δ = 126.20 (C_a_), 119.41 (C_b_), 120.07 (C_c_), 130.24 (C_d_), 143.47 (C_e_), 160.61 (C_f_), 159.18 (C_g_), 136.89 (C_h_).

##### 4,4′-((Sulfonylbis (4,1-phenylene)) bis(oxy)) dianiline (BAPS)

IR (KBr, cm^−1^): 3455, 3365, 1580 (N-H stretching), 1231 (C-O-C stretching). ^1^H NMR (500 MHz, DMSO-d_6_): 6.64 (4H, H_a_), 6.80 (4H, H_b_), 6.98 (4H, H_c_), 7.84 (4H, H_d_), 5.10 (4H, H_e_). ^13^C NMR (500 MHz, DMSO-d_6_): δ = 121.60 (C_a_), 115.12 (C_b_), 116.63 (C_c_), 129.72 (C_d_), 144.00 (C_e_), 146.59 (C_f_), 163.16 (C_g_), 134.39 (C_h_).

##### 4,4′-((Propane-2,2-diyl) bis(4-nitrophenoxy) benzene) (BPA-DN)

IR (KBr, cm^−1^): 1342, 1514 (-NO_2_ stretching), 1245 (C-O-C stretching). ^1^H-NMR (500 MHz, DMSO-d_6_): 8.22 (4H, H_a_), 7.10 (4H, H_b_), 7.07 (4H, H_c_), 7.35 (4H, H_d_). ^13^C NMR (500 MHz, DMSO-d_6_): δ = 126.57 (C_a_), 117.72 (C_b_), 120.34 (C_c_), 129.19 (C_d_), 30.97 (C_e_), 142.64 (C_f_), 163.31 (C_g_), 152.62 (C_h_), 147.58 (C_i_), 40.36 (C_j_).

##### 4,4′-(Propane-2,2-diylbis(4,1-phenylene)) bis(oxy)) dianiline (BAPP)

IR (KBr, cm^−1^): 3401, 3331, 1610 (N-H stretching), 1217 (C-O-C stretching). ^1^H NMR (500 MHz, DMSO-d_6_): 6.64 (4H, H_a_), 6.63 (4H, H_b_), 7.10 (4H, H_c_), 7.11 (4H, H_d_), 1.57 (4H, H_e_). ^13^C NMR (500 MHz, DMSO-d_6_): δ = 120.99 (C_a_), 116.08 (C_b_), 118.00 (C_c_), 127.74 (C_d_), 30.82 (C_e_), 143.88 (C_f_), 145.39 (C_g_), 156.83 (C_h_).

### 3.2. Polyimide Film Synthesis

**Figure 5:**
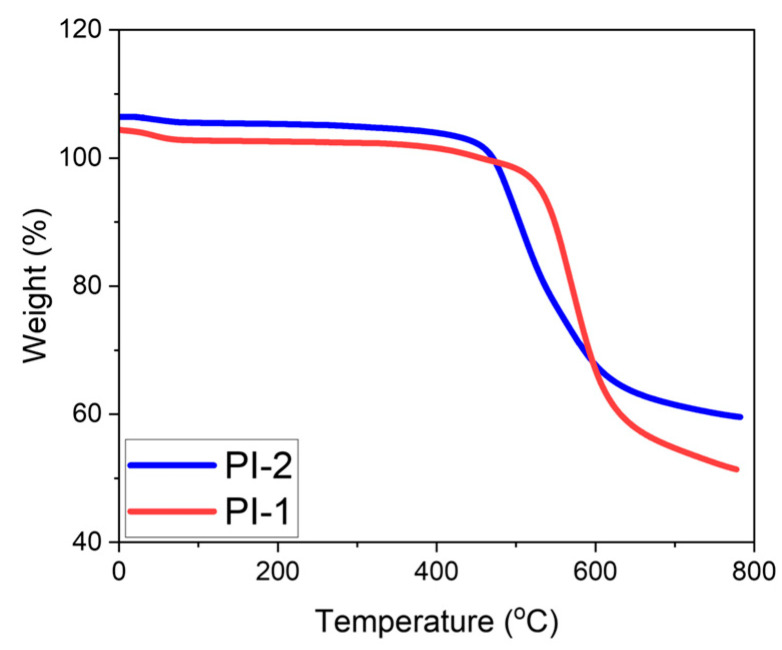
TGA Results of Polyimide Films. (Note: The initial mass slightly exceeds 100% due to instrument auto-normalization).

PAAs were converted into PI films using a thermal imidization procedure. The resins were cast onto cleaned aluminum plates. They were then heated at 80 °C for 1 h, 120 °C for 1 h, 150 °C for 1 h, 220 °C for 1 h, and 300 °C for 1 h. Upon completion, the films were removed from the aluminum plates. TGA plots of films are shown in Figure 5.

Figure 5 shows the thermogravimetric analysis results that were presented for the synthesized PIs. So, the BPS-based PI film exhibited thermal decomposition behavior at a higher temperature than the BPA-based PI film. Considering the chemical structures of the monomers, the -SO_2_-group attracts more electrons to the benzene ring, increasing the dipole–dipole bond interactions in the bps-based sample structure [36]. Consequently, the sample begins to decompose at a higher temperature. In the BPA-based PI film, the isopropylidene bridge of the monomer provides flexibility, and shows lower thermal resistance.

**Figure 6:**
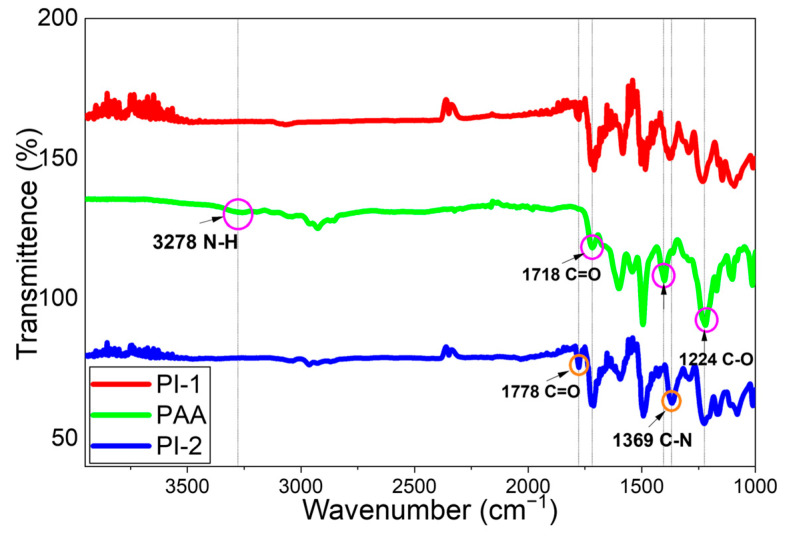
FTIR Results of PAA and PI Films.

The FTIR spectra of the PAA and PI samples are shown in Figure 6. According to Figure 6, the peak observed around 3278 cm^−1^ belongs to the ammonium groups, the peak observed at 1718 cm^−1^ belongs to the aromatic carboxyl group (C=O), the amide carbonyl group at 1660 cm^−1^, the N–H, C–N stretches at 1404 cm^−1^ and C-O stretches at 1224 cm^−1^. After gradual heating, the polyamic acid transforms into polyimide. This transformation is confirmed by the peaks in the PI spectrum belonging to the aromatic imide carbonyl group at approximately 1778 cm^−1^. On the other hand, the increasing of the C–N bonds grow with the peak wavenumber at 1369 cm^−1^, confirming the complete transformation of PAA to polyimide [15,44,45,46].

### 3.3. Characterization of Composite Materials

#### 3.3.1. Morphological Analysis

**Figure 7:**
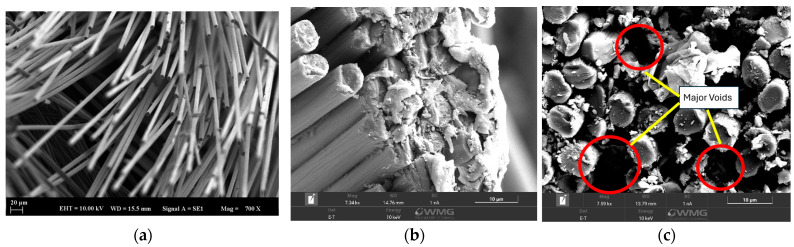
SEM Images of CF-PEEK-PI Composites (**a**) Bare CF-PEEK (**b**) CF-PEEK-PI-1 (**c**) CF-PEEK-PI-2.

Scanning electron microscopy (SEM) analysis was performed on the materials obtained after tensile testing (Figure 7). A comparison was made with a material without any sizing agent to check the coating. As seen in Figure 7a, the fiber surfaces in the desizing composites are bare and this can be attributed to the weak interaction at the fiber–matrix interface. In contrast, in the sizing composites (Figure 7b, c), the fibers were coated with matrix, and matrix particles were seen to adhere to the fibers at the fracture surfaces. Despite the presence of voids (red circle in Figure 7c), sizing generally improves adhesion at the fiber–matrix interface.

#### 3.3.2. Thermal Properties

**Figure 8:**
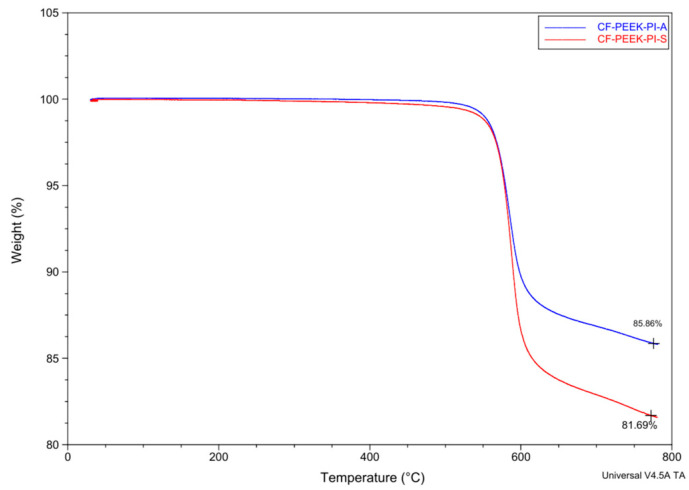
TGA Analysis of CF-PEEK-PI-A and CF-PEEK-PI-S Composites.

Figure 8 presents thermogravimetric behavior of CF-PEEK-PI-A and CF-PEEK-PI-S composites materials. Because both materials (CF-PEEK-PI-A and CF-PEEK-PI-S) have aromatic groups in their structures, they are expected to be resistant to thermal degradation up to around 500–600 °C (Figure 8). The CF-PEEK-PI-A composite leaves 85.86% residue, while the CF-PEEK-PI-S composite leaves 81.69% residue at 800 °C. This difference is attributed to the higher polarity of the CF-PEEK-PI-S structure, which accelerates the initial bond cleavage within the polymer matrix. The residue levels show both the polymer matrix degradation and the effect of the polyimide sizing agent.

**Figure 9:**
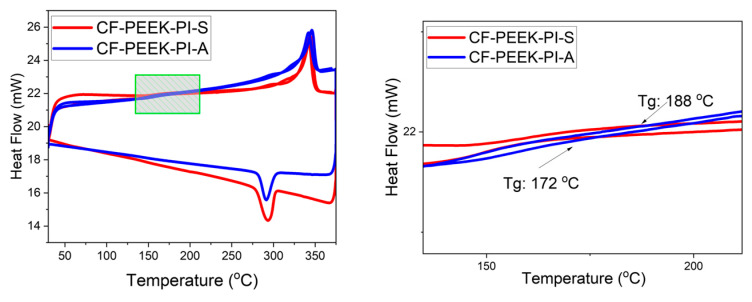
DSC Analysis of CF-PEEK-PI-A and CF-PEEK-PI-S Composites.

The Differential Scanning Calorimetry (DSC) graph is shown in Figure 9. The crystallinity values of composite materials based on DSC graphs were calculated using the following formula [47]. ΔHm: Measured melting enthalpy; ΔHm^0^: Melting enthalpy of 100% crystalline PEEK; w: PEEK ratio in the composite.

(1) $$\mathrm{Formulation}\mathrm{of}\mathrm{Crystallinity}\mathrm{Degree}\;Xc\left(\%\right)=\frac{\Delta \mathrm{Hm}}{\Delta {\mathrm{Hm}}^{0}}\times 100$$

As shown in Figure 9, the degree of crystallinity of the CF-PEEK-PI-A sample was calculated to be approximately 23.4%. In contrast, this value increased to 31.1% in the CF-PEEK-PI-S composite. According to the results, the sulfonyl group in the structure of the CF-PEEK-PI-S sample makes the structure more polar and provides a more ordered crystalline structure in the polymer chain. In contrast, the lower crystallinity value of the CF-PEEK-PI-A sample can be attributed to the flexibility restriction of the isopropylidene bridge present in the structure. Figure 9 includes a magnified view of the region highlighted in the left figure to clearly display the glass transition temperatures (Tg) of the CF-PEEK-PI-A and CF-PEEK-PI-S samples. Table 2 presents the DSC results, including Tg, Tm, ΔH and crystallinity values for both samples.

Dynamic Mechanical Analysis (DMA) provides information about the thermomechanical behavior and energy dissipation capacity between the reinforcement element and the matrix. The storage modulus (E′) is a parameter that quantitatively expresses the elastic response of a material, the energy stored during elastic deformation [48]. In contrast, the loss modulus (E″) gives detailed information about the material’s viscous properties [49]. Tan δ is a component that expresses the balance between the elastic and viscous components. Tan delta is calculated by dividing the loss modulus by the storage modulus [49,50,51,52,53,54]. A higher value indicates that the material is more viscous and has a higher energy dissipation capacity, while the opposite value indicates that the material has a high energy storage capacity and is elastic [48].

**Figure 10:**
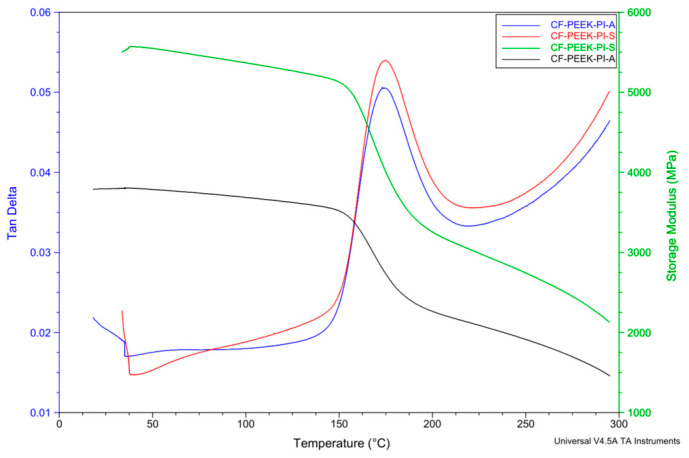
DMA Analysis of CF-PEEK-PI-A and CF-PEEK-PI-S Composites.

According to the tan δ curves (Figure 10), the glass transition temperatures (Tg) of the CF-PEEK-PI-A and CF-PEEK-PI-S composites were found to be approximately 173 °C and 174 °C, respectively. This result indicates that the use of different monomers does not make a significant difference in Tg. However, when comparing the storage modulus values, it can be said that the CF-PEEK-PI-S composites have higher initial stiffness compared to the CF-PEEK-PI-A composites. Because sulfonyl groups make chain movement more difficult, resulting in a higher storage modulus. Therefore, the CF-PEEK-PI-S material is expected to have a higher storage modulus.

The Tg values obtained from DSC (Table 2) were in reasonable agreement with the tan delta values observed in DMA, especially for the CF-PEEK-PI-A sample; however, the slightly higher Tg of CF-PEEK-PI-S in DSC may be attributable to differences in the measurement principles of the two techniques.

#### 3.3.3. Mechanical Properties

**Figure 11:**
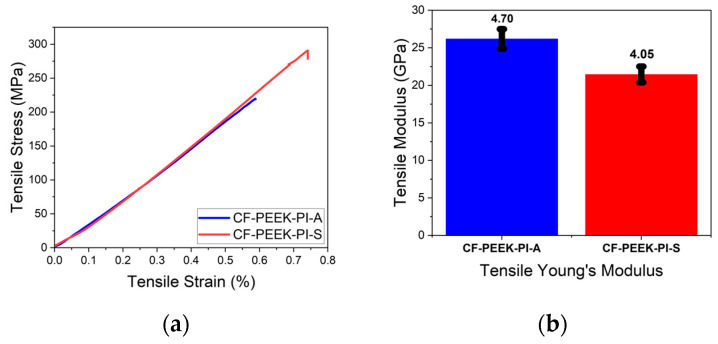
Tensile (**a**) and Tensile Modulus (**b**) Curves for CF-PEEK-PI Composites.

From the obtained tensile test result (Figure 11), the CF-PEEK-PI-S sample showed higher tensile strength (288 ± 10 MPa) than the CF-PEEK-PI-A sample (223 ± 39.1 MPa). In the literature, Gao et al. [14], reported a tensile strength of approximately 795 MPa for CF/PEEK composites process by hot-pressing without sizing agent. This tensile value can be attributed to the differences in the manufacturing process. On the other hand, CF-PEEK-PI-A sample showed superior properties with its elastic modulus value of 26.2 ± 4.70 GPa, which was higher than the CF-PEEK-PI-S sample, which had a value of 21.4 ± 4.05 GPa.

**Figure 12:**
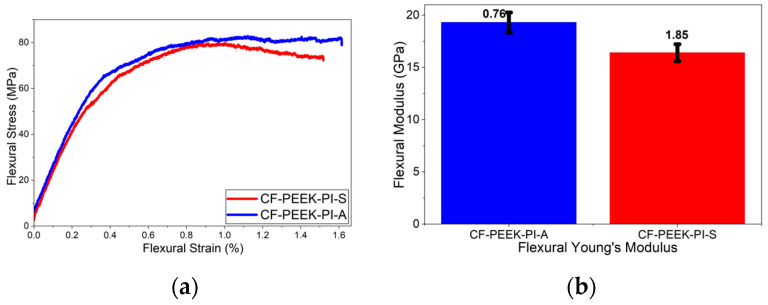
Flexural (**a**) and Flexural Modulus (**b**) Curves for CF-PEEK-PI Composites.

Based on the flexural test results (Figure 12), the CF-PEEK-PI-A composite, with values of 89.5 ± 7.56 MPa and 19.3 ± 0.76 GPa, exhibited higher performance compared to the CF-PEEK-PI-S composite, with values of 73.5 ± 5 MPa and 16.4 ± 1.85 GPa, in terms of both flexural strength and flexural modulus. According to a study by Ren et al. [54], CF/PEEK composites produced without sizing agents show flexural strengths of approximately 410 MPa, while modified CF/PEEK composites can reach up to 690 MPa depending on surface treatment parameters. Furthermore, flexural modulus values for materials without any sizing agents have been reported to be 21 GPa.

**Figure 13:**
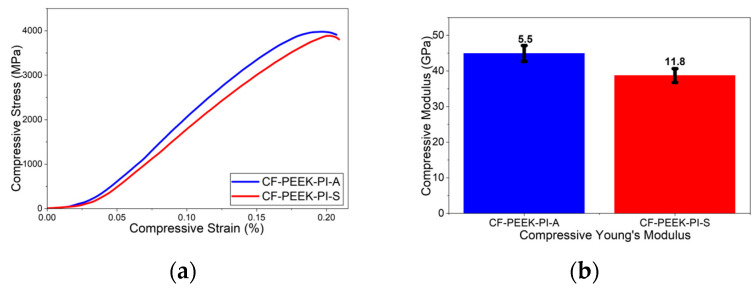
Compressive (**a**) and Compressive Modulus (**b**) Curves for CF-PEEK-PI Composites.

The compressive test result showed that (Figure 13) the CF-PEEK-PI-A composite exhibits higher both compressive strength (53.1 ± 5.38 MPa) and compressive modulus (44.9 ± 5.5 GPa). However, the CF-PEEK-PI-S composite showed values of 50.1 ± 6.6 MPa and 38.7 ± 11.8 GPa, respectively. In the study conducted by Shang-Lin et al. [51], they reported the compressive test strength of CF/PEEK composite materials as approximately 140 MPa and the compressive modulus value as 4.5 GPa.

**Figure 14:**
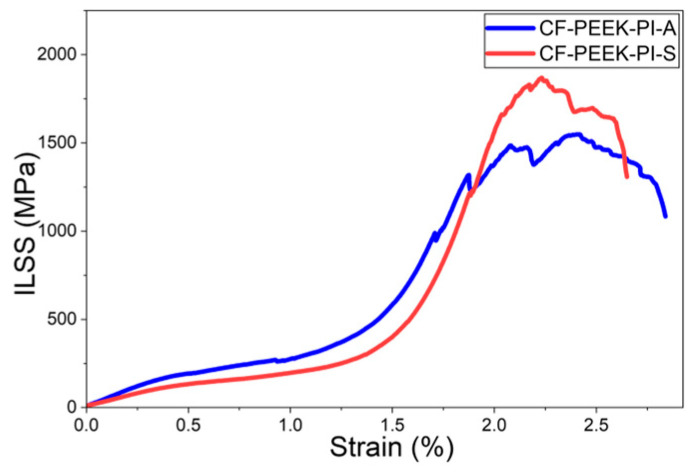
ILSS Curves for CF-PEEK-PI Composites.

Lastly, according to the Interlaminar Shear Strength (ILSS) results (Figure 14), the CF-PEEK-PI-S composite showed better performance than the CF-PEEK-PI-A composite (93.4 ± 8.65 MPa) with a value of 119 ± 7.67 MPa. In the literature, ILSS values of CF/PEEK composites have been reported to be in the range of approximately 55–60 MPa [14].

According to comparison results with literature data, the overall tensile and flexural strength values obtained in this study are lower than those reported in the literature. This can be attributed to differences in fiber wetting efficiency with different processing methods. However, the ILSS values are comparable to previously reported CF/PEEK composites, demonstrating that PI-based sizing agents are effective in improving fiber–matrix interfacial bonding despite the limitations of processing conditions.

Comer et al. [55], reported flexural strength and ILSS values for fully impregnated commercial CF/PEEK prepreg systems processed in an autoclave. Accordingly, the flexural strength was 1650 MPa and the ILSS was 94 MPa. The relatively high flexural strength can be attributed to the fact that the prepreg strips used provided extremely low void ratios and were produced under industrial conditions. Furthermore, the powdered PEEK used in the present study was found to result in higher void ratios when high-efficiency impregnation was not achieved, leading to lower-than-expected mechanical test results.

Therefore, mechanical properties should not be compared directly with high-performance prepregs but rather evaluated in the context of powder-based CF/PEEK composites. In this category, the PI-S modification shows significant improvement in ILSS, indicating increased interfacial interaction.

## 4. Conclusions

In this study, PI-based sizing agents were produced using two different bisphenol-based diamine monomers (bisphenol-A and bisphenol-S) to improve the fiber–matrix interface in carbon fiber-reinforced PEEK (CF/PEEK) composites. The resulting sizing agents were coated onto the CF surface via thermal imidization, producing composites with two distinct properties. DMA analyses indicate that the CF-PEEK-PI-S composites have a higher storage modulus (E′), which is related to the mechanical strength provided by the sulfone group.

According to mechanical test results, the CF-PEEK-PI-S sample performed better in both tensile (288 MPa) and ILSS (119 MPa) tests than the BPA-based composite while the CF-PEEK-PI-A composite achieved higher results in both flexural and compression tests, than the BPS-based composite, with values of 89.5 MPa and 53.1 MPa, respectively.

Overall, it was determined that BPS-based polyimide exhibits superior tensile and shear strength because of its strong interfacial interactions. BPA-based polyimide may contribute to slightly improved flexural and compressive behavior, possibly due to enhanced interfacial adhesion instead of intrinsic/structural flexibility.

This study evaluated the impact of chemically different groups (isopropylidene and sulfone) on the interfacial behavior of thermoplastic composite materials which provide a guiding basis for future high-performance composite production.

## Figures and Tables

**Table 1:** Chemical Structures of Polyimide Monomers.

Diamine	Dianhydride	Resin	Polyimide
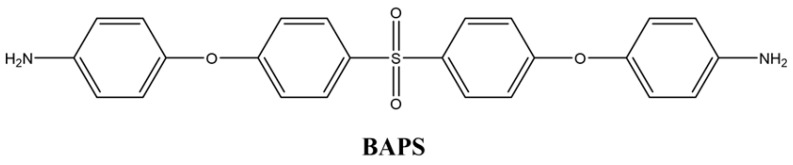	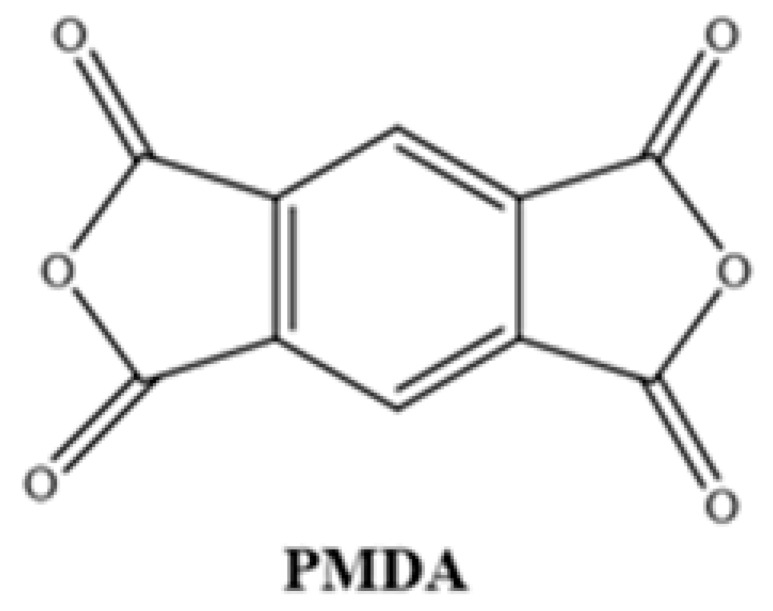	PAA-1	PI-1
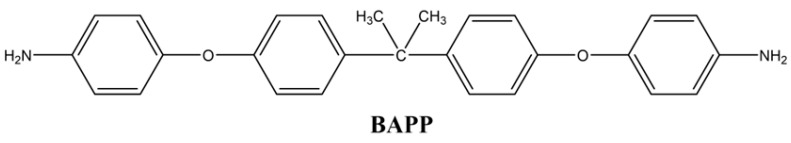	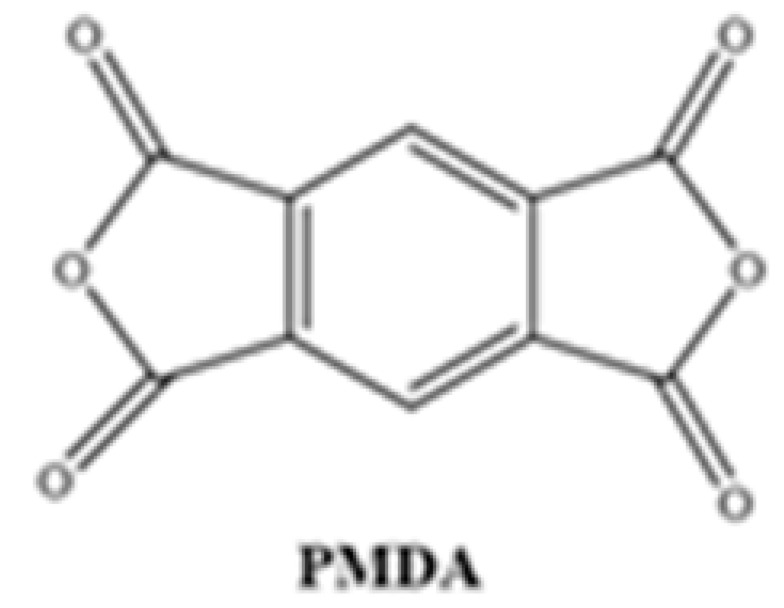	PAA-2	PI-2

**Table 2:** DSC analysis results for CF-PEEK-PI materials.

Sample	Tg (^o^C)	Tm (^o^C)	∆H_c_ (J/g)	Crystallinity (%)
CF-PEEK-PI-A	172	291	−12.16	23.4
CF-PEEK-PI-S	188	293	−16.18	31.1

## Data Availability

The original contributions presented in this study are included in the article/Supplementary Material. Further inquiries can be directed to the corresponding author.

## Supplementary Materials

The following supporting information can be downloaded at: https://www.mdpi.com/article/10.3390/polym17243258/s1, The full FTIR spectra, DSC curves, 1H and 13C NMR spectra for the synthesized monomers of polyimides are provided as Supplementary Materials. Figure S1: FTIR Spectrum of DNPSB. Figure S2: FTIR Spectrum of BAPS. Figure S3: FTIR Spectrum of MeP-BOBNB. Figure S4: FTIR Spectrum of BAPP. Figure S5: DSC Curve of DNPSB. Figure S6: DSC Curve of BAPS. Figure S7: DSC Curve of BPA-DN. Figure S8: DSC Curve of BAPP. Figure S9: 1H NMR spectrum of DNPSB. Figure 10: 13C NMR spectrum of DNPSB. Figure 1: 13C NMR spectrum of DNPSB. Figure S12: 13C NMR spectrum of BAPS. Figure S13: 1H NMR spectrum of BPA-DN. Figure S14: 13C NMR spectrum of BPA-DN. Figure S15: 1H NMR spectrum of BAPP. Figure S16: 13C NMR spectrum of BAPP.

## Abbreviations

The following abbreviations are used in this manuscript:

BPA	Bisphenol A
BPS	Bisphenol S
CF	Carbon Fiber
DMA	Dynamic Mechanical Analysis
DSC	Differential Scanning Calorimetry
FTIR	Fourier Transform Infrared Spectroscopy
ILSS	Interlaminar Shear Strength
PAA	Poly (amic acid)
PEEK	Poly (ether ether ketone)
PI	Polyimide
SEM	Scanning Electron Microscopy
TGA	Thermogravimetric Analysis
