# Supplementary material for: Enhancing Interfacial and Mechanical Properties of Carbon Fiber/Poly (Ether Ether Ketone) Composites via Bisphenol-Based Polyimide Modification
Source: Polymers (Basel). 2025 Dec 7;17(24):3258. doi: 10.3390/polym17243258 (PMC12736752; doi:10.3390/polym17243258)
Supplement: Supplementary file 1 [file polymers-17-03258-s001.zip › polymers-3981690-supplementary.pdf]

Enhancing Interfacial and Mechanical Properties of Carbon Fiber/Poly (Ether Ether Ketone) Composites via Bisphenol-Based Polyimide Modification

Aylin Albayrak[1*], Mustafa Dogu[2], Mustafa Cakir[3] and Kadir Turhan[1]

[1] Department of Chemistry, Faculty of Arts and Sciences, Yildiz Technical University, Davutpasa Campus, Esenler, Istanbul, Türkiye; aylin.albayrak@std.yildiz.edu.tr; kturhan@yildiz.edu.tr

[2]Research Department, Mir Arastirma ve Gelistirme Inc., Esenyurt, Istanbul, Türkiye; mus.dogu@gmail.com

[3]Department of Materials and Metallurgical Engineering, Marmara University, Istanbul, Türkiye; mcakir@marmara.edu.tr

* Correspondence: aylin.albayrak@std.yildiz.edu.tr; aylin.albayrak1@outlook.com

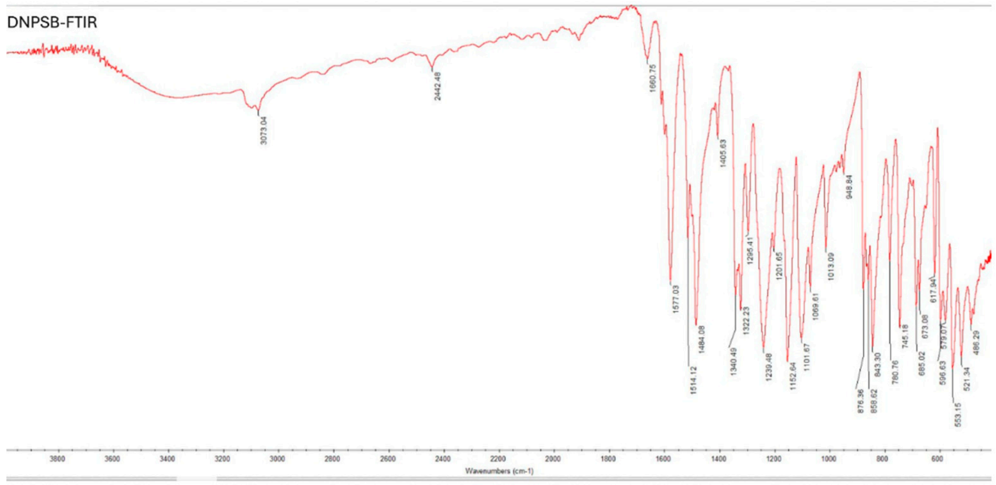

Figure S1 FTIR Spectrum of DNPSB

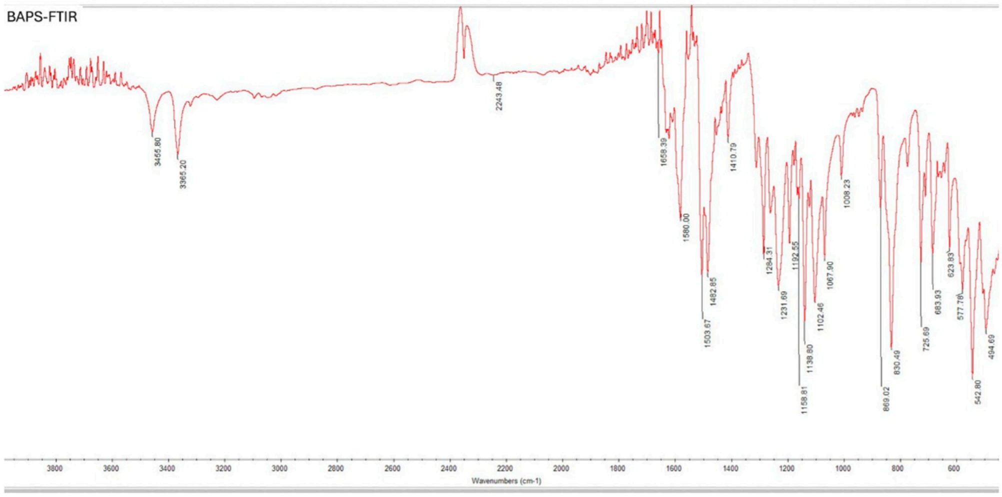

Figure S2 FTIR Spectrum of BAPS

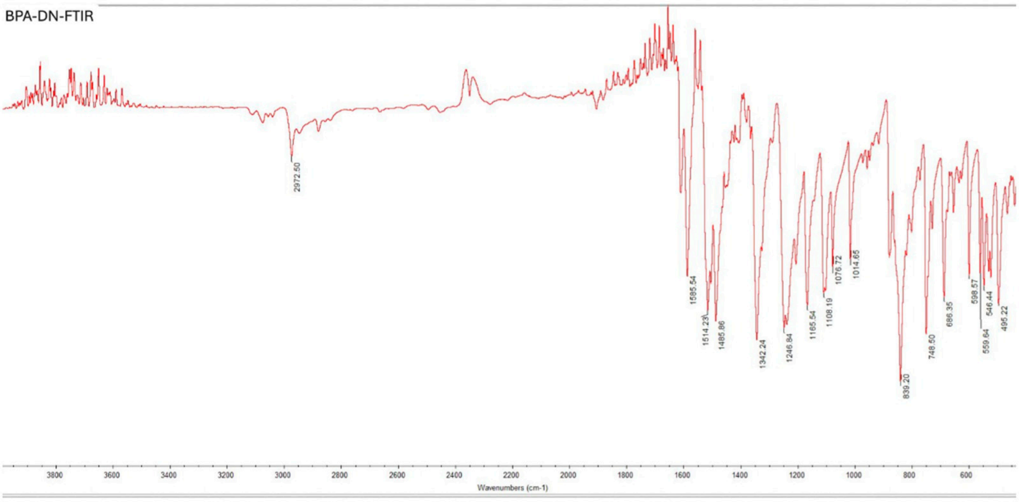

Figure S3 FTIR Spectrum of MeP-BOBNB

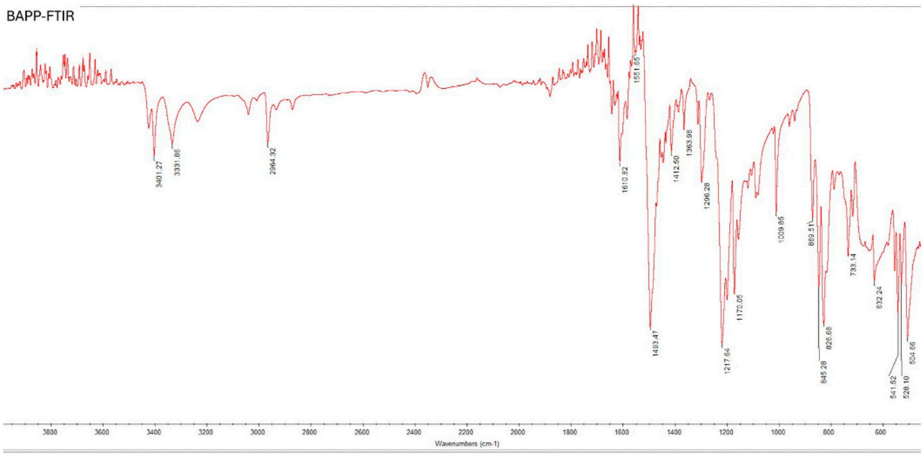

Figure S4 FTIR Spectrum of BAPP

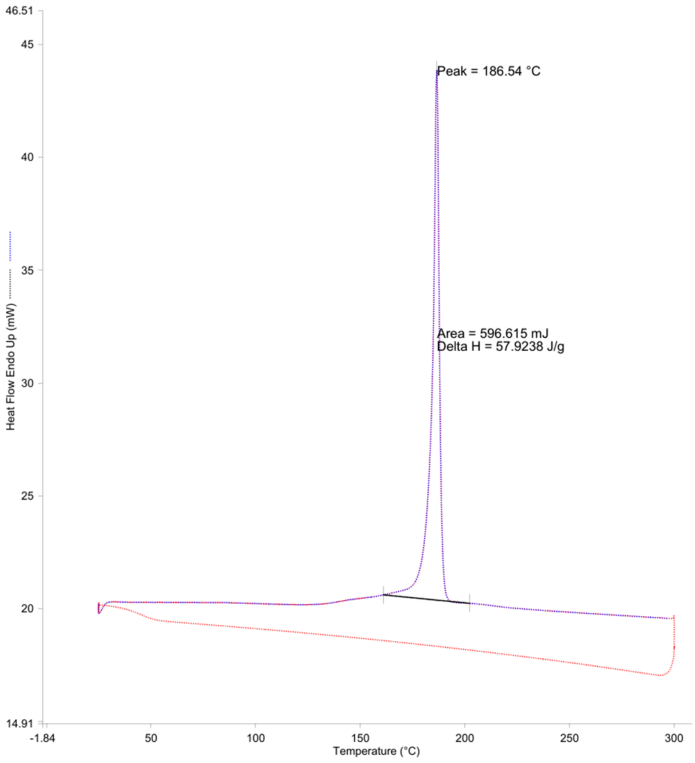

Figure S5 DSC Curve of DNPSB

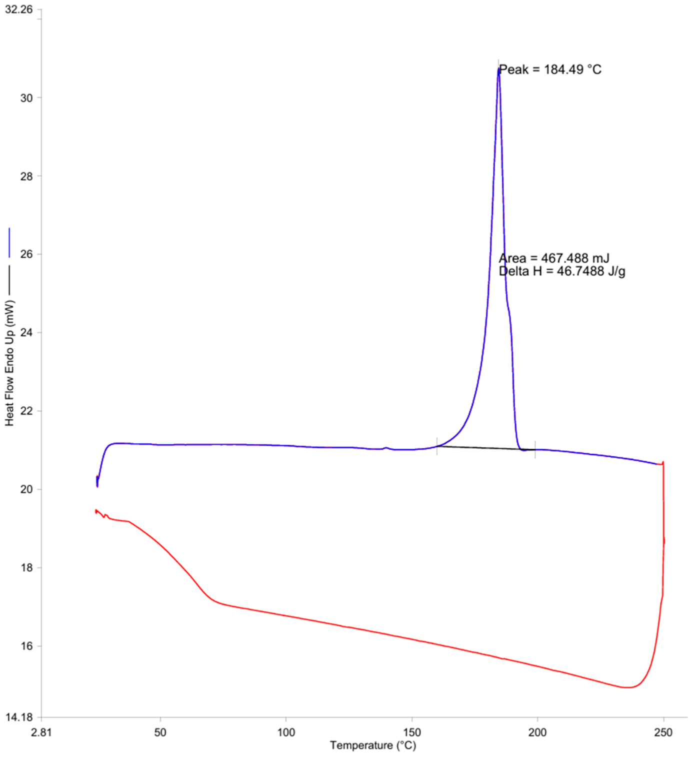

Figure S6 DSC Curve of BAPS

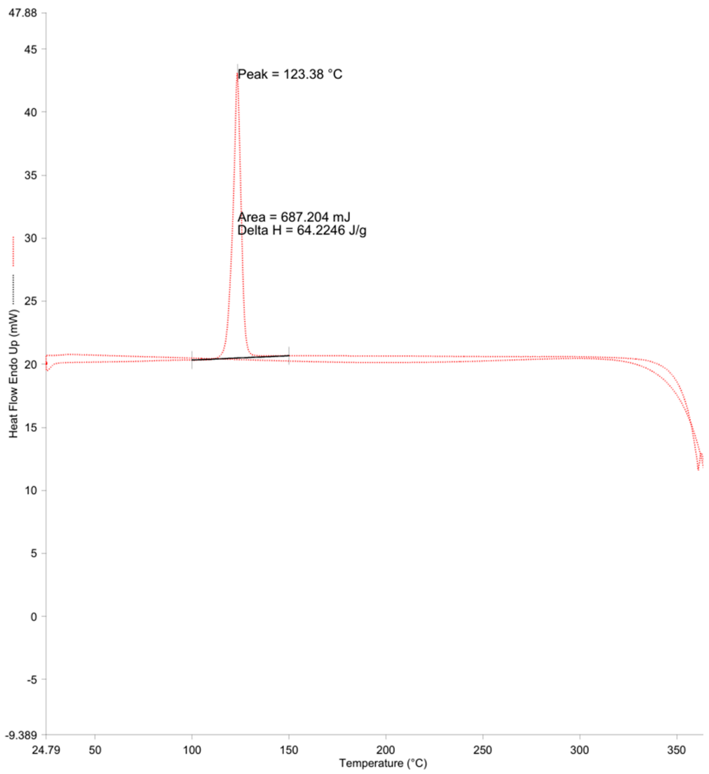

Figure S7 DSC Curve of BPA-DN

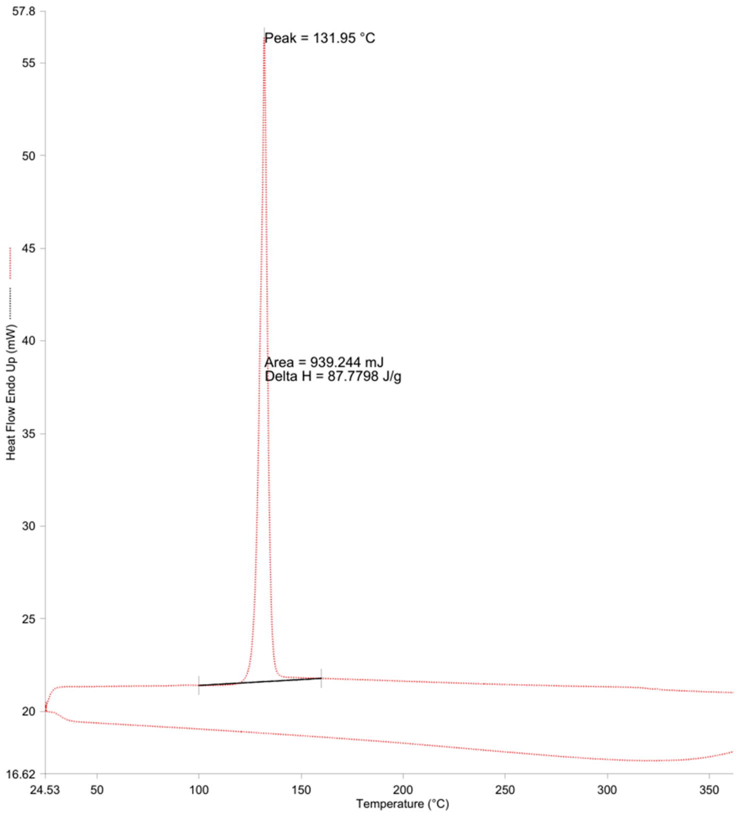

Figure S8 DSC Curve of BAPP

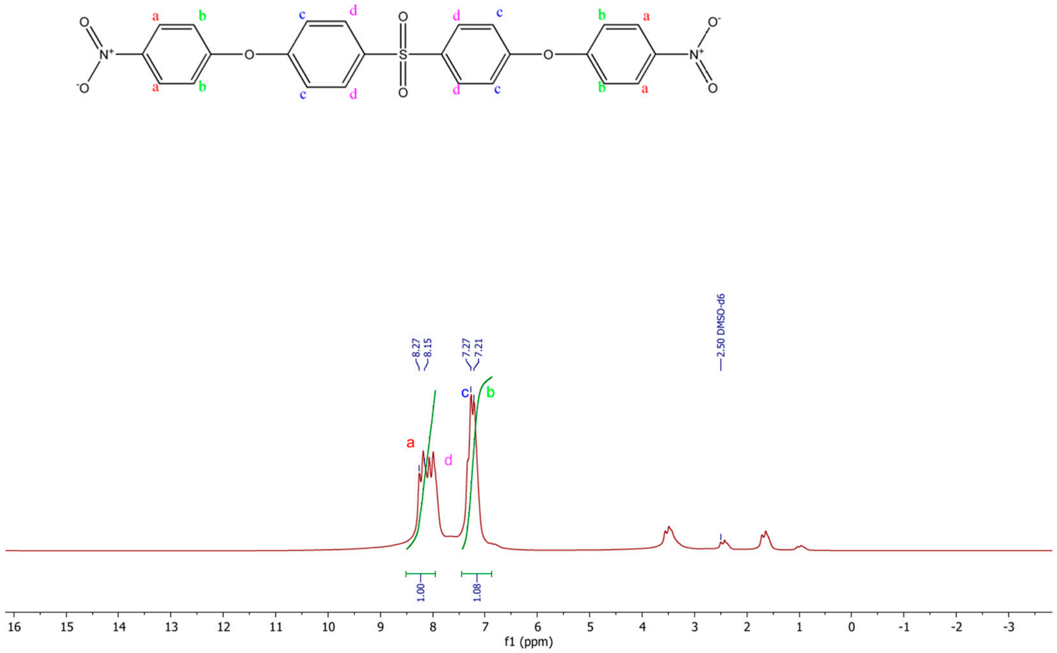

Figure S9 ^{1}H NMR spectrum of DNPSB

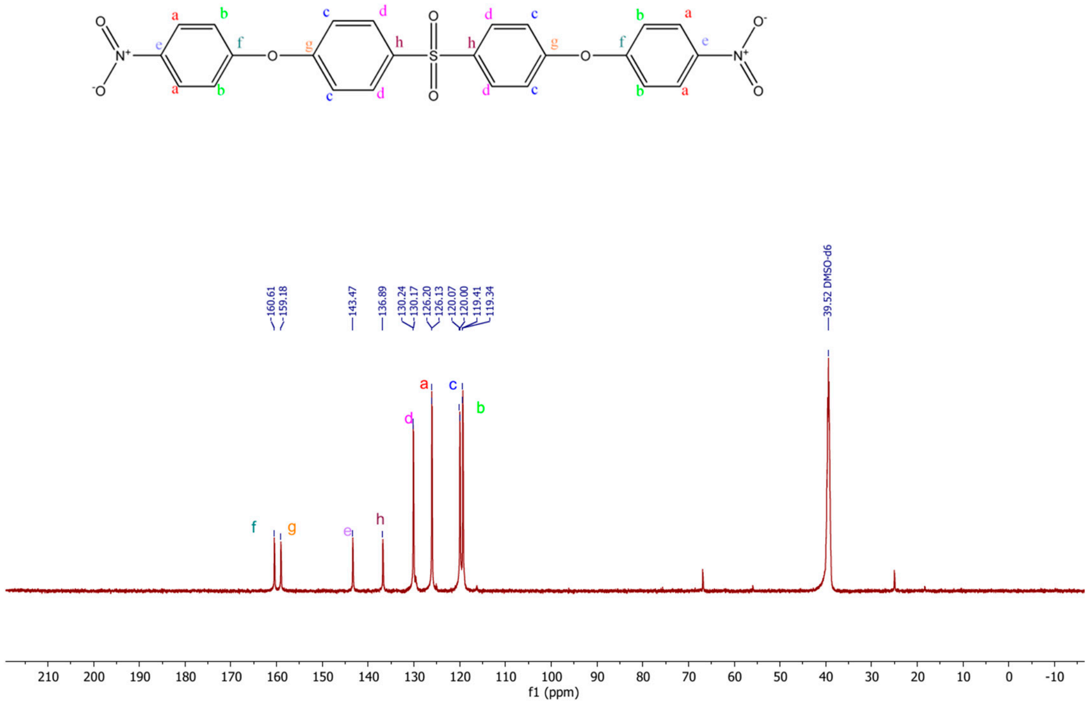

Figure 10 ^{13}C NMR spectrum of DNPSB

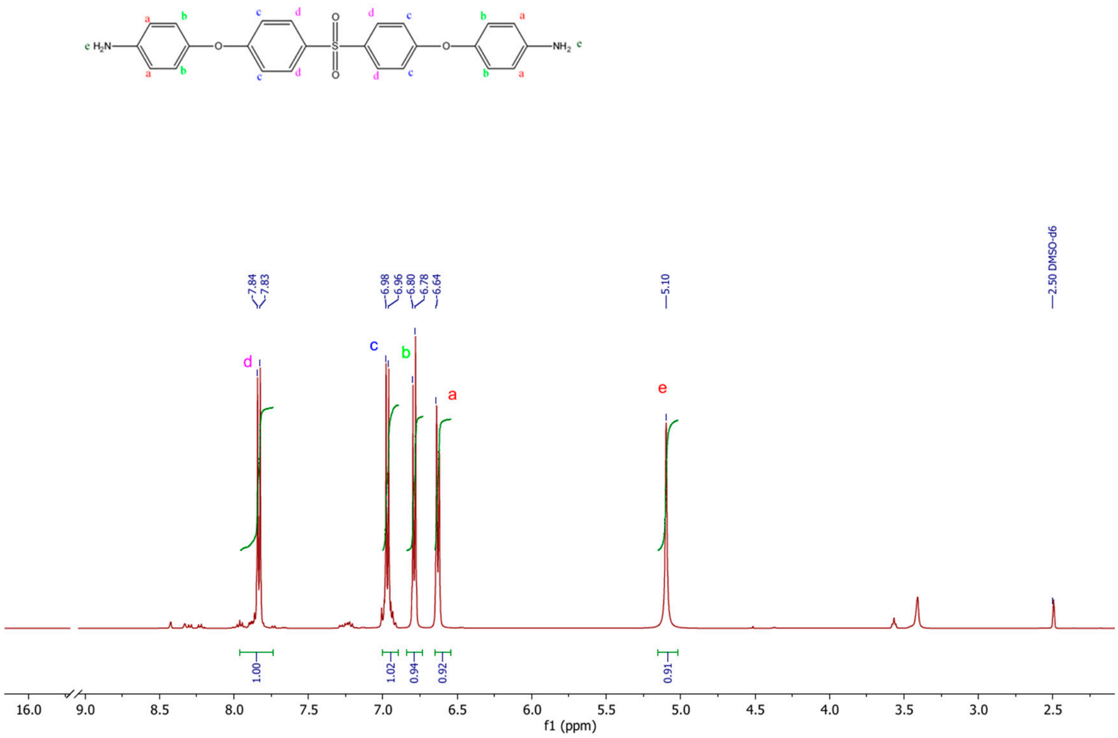

Figure S11 ^{1}H NMR spectrum of BAPS

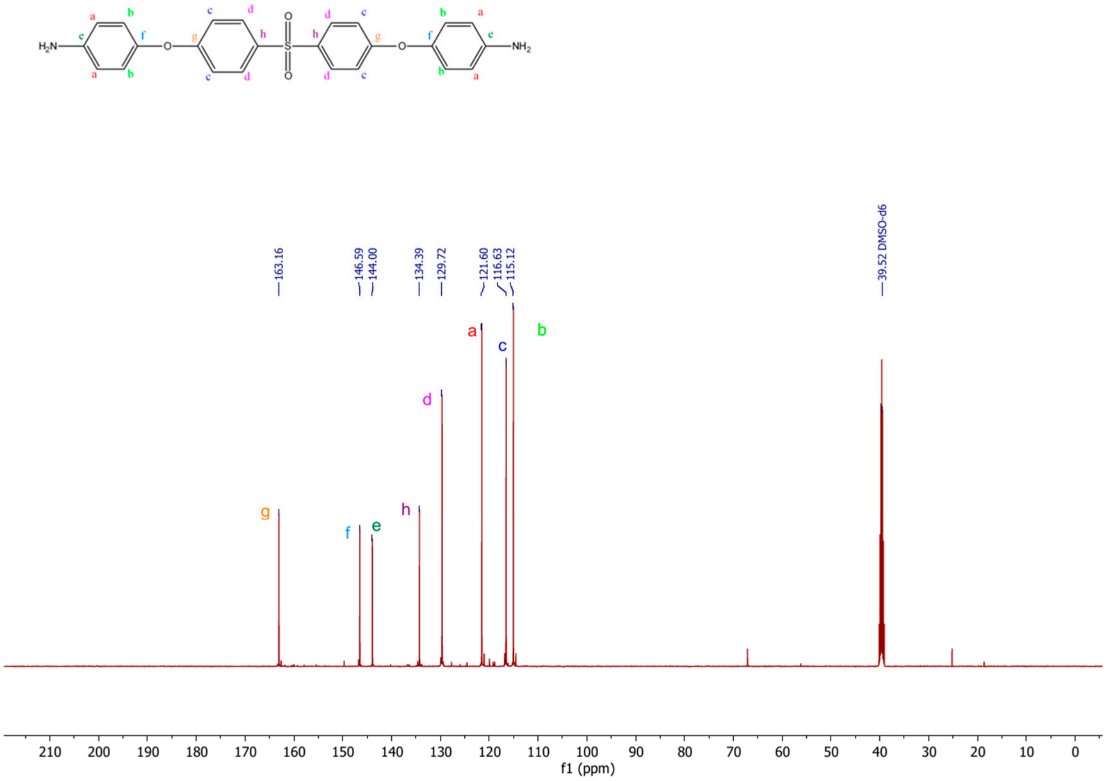

Figure S12 ^{13}C NMR spectrum of BAPS

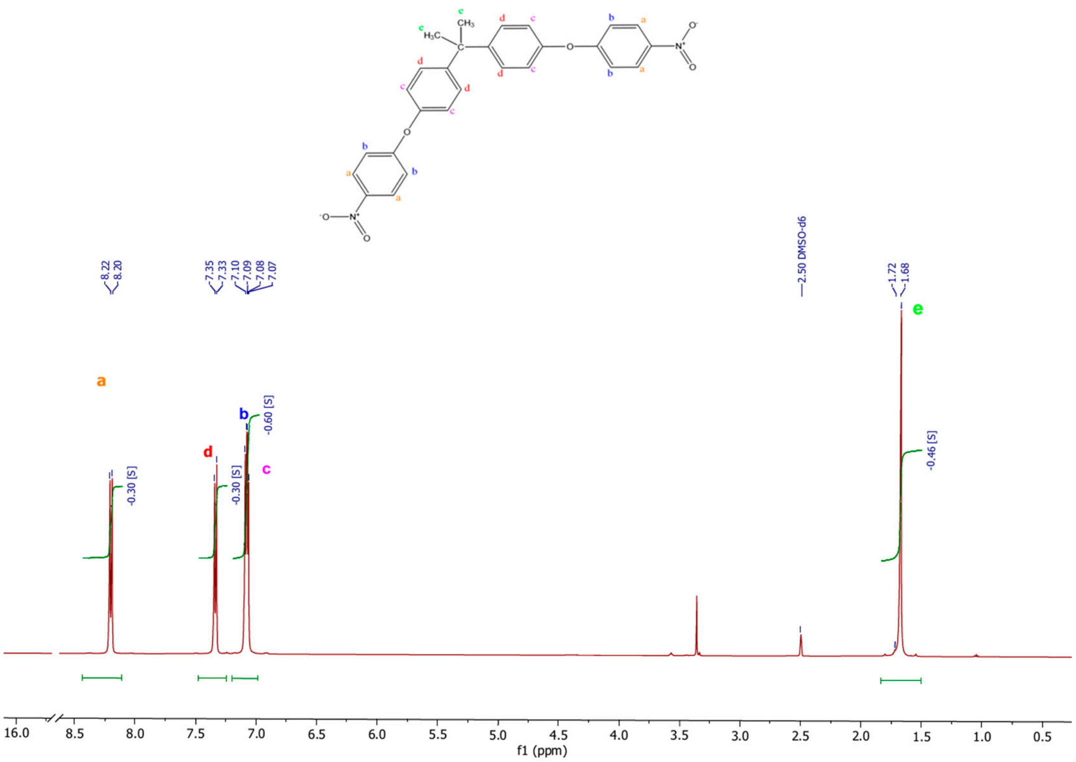

Figure S13 ^{1}H NMR spectrum of BPA-DN

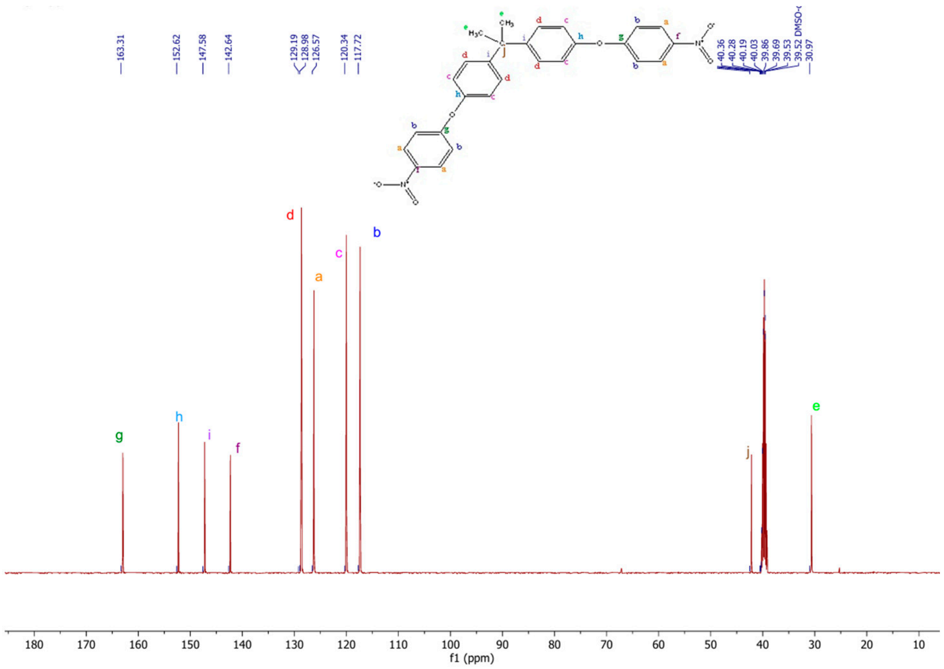

Figure S14 ^{13}C NMR spectrum of BPA-DN

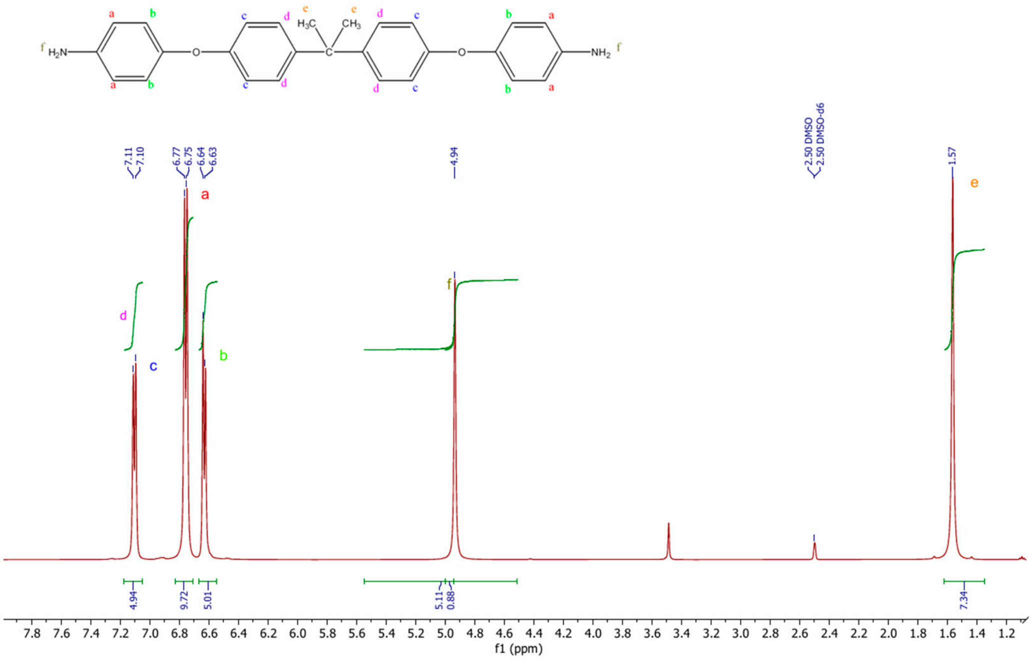

Figure S15 ^{1}H NMR spectrum of BAPP

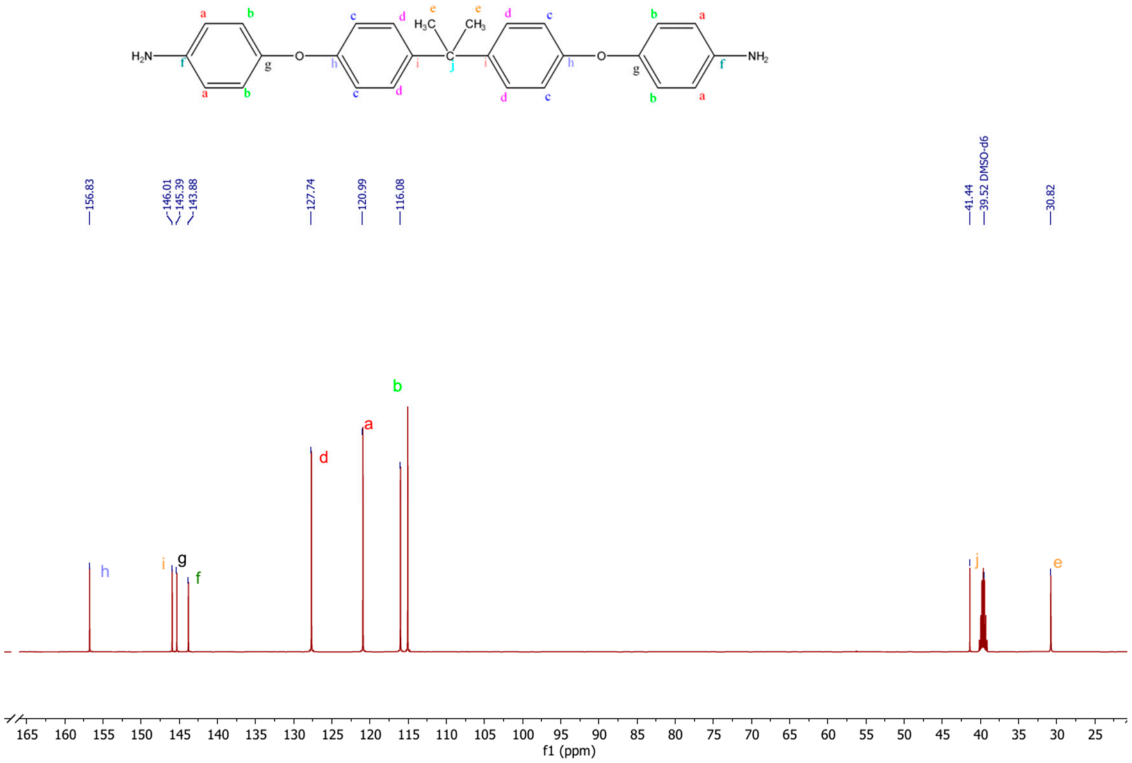

Figure S16 ^{13}C NMR spectrum of BAPP
